# Purastat as an Adjunct Treatment Option in Acute Esophageal Varices Bleeding: A Case Report

**DOI:** 10.7759/cureus.42712

**Published:** 2023-07-30

**Authors:** Imran H Hassan, David Elphick, Ammar Al-Rifaie

**Affiliations:** 1 Gastroenterology, Chesterfield Royal Hospital, Chesterfield, GBR

**Keywords:** esophageal varices, cirrhosis liver, complications of cirrhosis, acute gastrointestinal bleed, purastat®

## Abstract

Esophageal varices are dilated submucosal esophageal veins that connect the portal and systemic circulations. Bleeding esophageal varices is a well-recognized complication of liver cirrhosis.It is known that in active variceal bleeding, treatment needs to be started promptly. Treatments comprise band ligation, sclerotherapy, removable stent placement, balloon tamponade, and transjugular intrahepatic portosystemic shunt (TIPS).We report a case in which hemodynamic stability can be maintained with the use of Purastat to control bleeding.

## Introduction

Acute variceal bleeding is a severe complication of liver cirrhosis, and it is a leading cause of mortality in patients with this condition [1]. Purastat, a novel synthetic somatostatin analog, has emerged as an effective treatment for hemodynamic stabilization in gastrointestinal bleeding [2].

There is emerging evidence of the increase in the use of Purastat in both upper and lower GI bleeding. A recent publication showed that it has been used in gastric antral vascular ectasia syndrome (GAVE) [3]. Another example of Purastat use is in a study of 21 patients with severe hemorrhagic radiation proctopathy where a significant improvement in bleeding after the application was noted [4]. Purastat is indicated for hemostasis of blood ooze in the parenchyma of solid organs, vascular anastomoses, and small blood vessels or capillaries of the GI tract [5].

In our case, we present another use of Purastat in post-esophageal banding with success. This could potentially increase the endoscopic tools used to control refractory esophageal variceal bleeding.

## Case presentation

A 67-year-old male was referred for a routine esophagogastroduodenoscopy (EGD) from primary care to our hospital due to epigastric bloating and discomfort despite taking 20 mg omeprazole daily and famotidine. His past medical history comprised of non-alcoholic fatty liver disease (NAFLD), type 2 diabetes mellitus, vitamin D deficiency, gastro-esophageal reflux disease, asthma, chronic obstructive pulmonary disease, and hypertension. For these conditions, he was on the following medications: candesartan 16 mg OD, Braltus 10 mcg OD, carbocisteine 750 mg TDS, empagliflozin 25 mg OD, fluoxetine 20 mg OD, Fobumix 160/4.5, omeprazole 20 mg OD, Salamol inhaler, and metformin 1.5 g OD. The patient denied any alcohol consumption and was an ex-smoker with a 40-year history of smoking.

**Figure 1:**
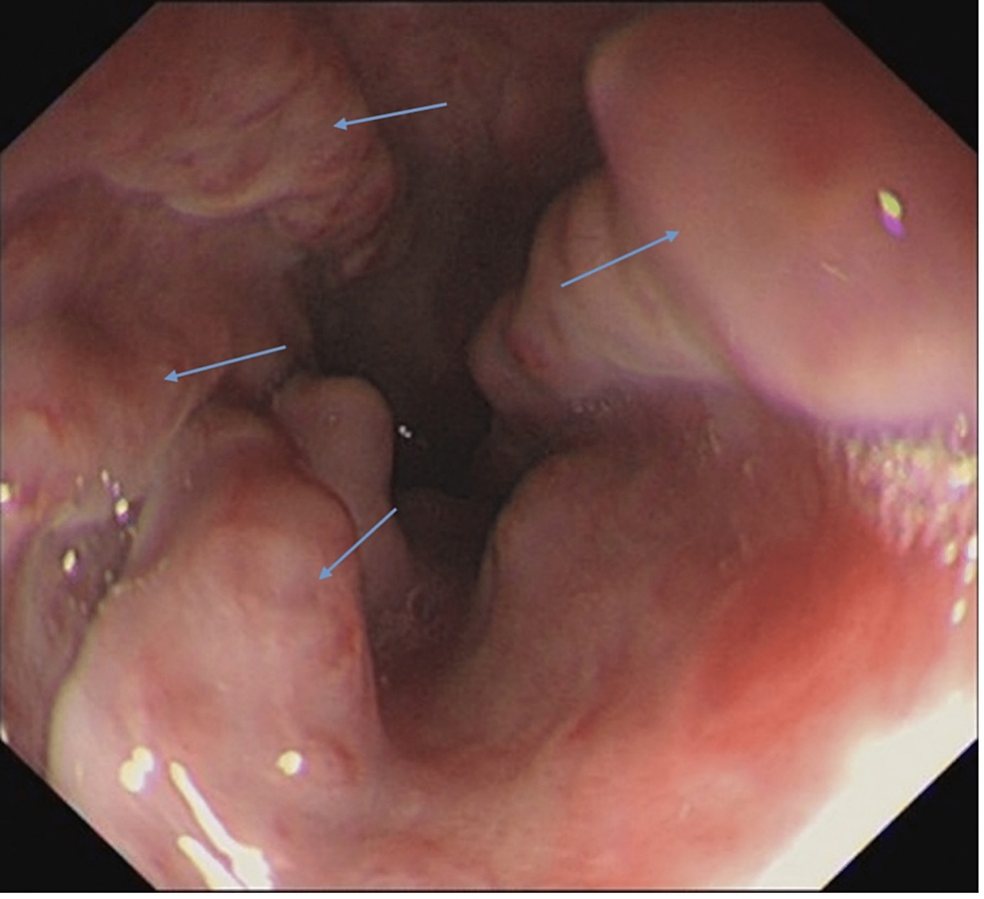
The initial EGD showing four columns of grade 2 esophageal varices with multiple red signs (blue arrows depicting the four columns) EGD, esophagogastroduodenoscopy

The EGD showed that the patient had four columns of grade 2 esophageal varices with multiple red signs and portal hypertensive gastropathy. Underlying NAFLD cirrhosis was suspected, and further investigation was arranged (Figure 1).

A non-invasive liver screen comprising blood testing for viral hepatitis B and C, autoimmune antibodies (antinuclear antibodies (ANA), anti-mitochondrial antibodies (AMA), and anti-liver-kidney microsomal (LKM) antibodies), alpha 1 anti-trypsin levels, iron studies, and immunoglobulins was done. These were all normal or negative. An ultrasound scan showed a cirrhotic liver with a splenomegaly of 13 cm. 

**Figure 2:**
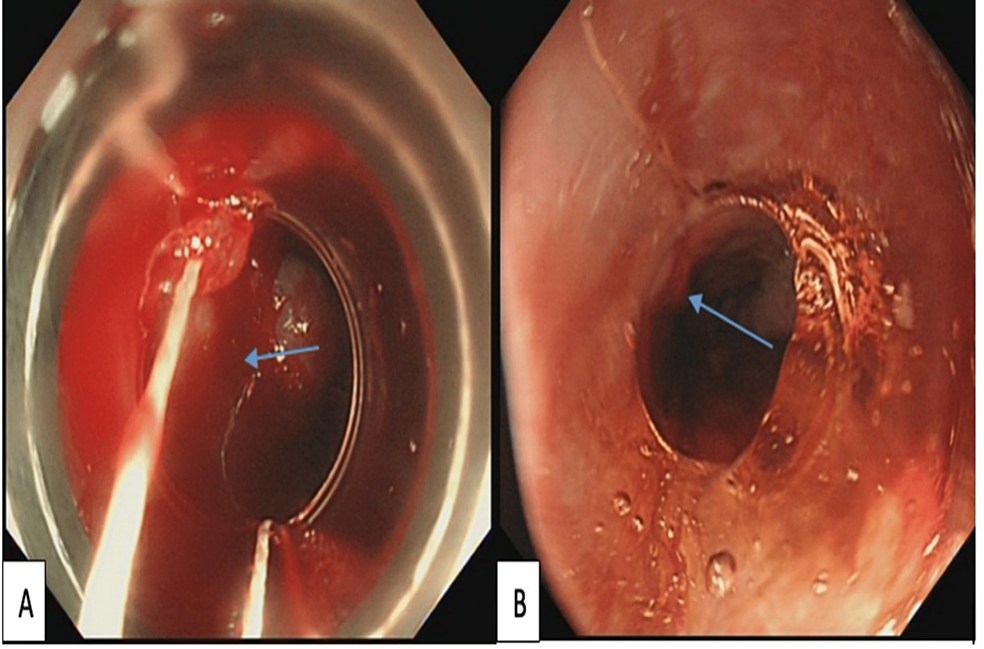
EGD for elective variceal banding. (A) Active bleeding from varices despite banding. (B) Successful resolution of bleeding after Purastat application EGD, esophagogastroduodenoscopy

Given the red signs on the varices at index EGD, a repeat procedure for elective variceal banding was arranged. Active bleeding started shortly after intubation with the bander on. Despite the application of one band at the bleeding varix and another between the bleeding varix and gastro-esophageal junction (GOJ), mild-to-moderate bleeding persisted. Further banding was applied on top of the same bleeding varix without success (Figure 2A). The bleeding continued for approximately five minutes, during which the oozing was significant enough to necessitate further intervention. 

During the intervention, various options were explored, including balloon tamponade, transjugular intrahepatic portosystemic shunt (TIPS), and Danis stent placement. Balloon tamponade was considered unnecessary as the patient remained hemodynamically stable throughout [6]. TIPS is not available locally. Danis stent placement is usually reserved for refractory cases with variceal bleeding when first-line endoscopic treatment fails. Danis stent was a valid option in our case [7].

A second endoscopy consultant attended, and a consensus decision was made to try applying Purastat to the bleeding area, reserving Danis stent for treatment failure. A trial of Purastat was initiated, with 6 ml successfully sprayed over the bleeding source and around the bands, with an endoscopic catheter (Purastat nozzle system type E). Upon application, Purastat immediately and effectively controlled the oozing, providing rapid and efficient hemostatic action (Figure 2B). To complete the procedure, the remaining three columns were banded with just one band each.

The patient was then kept under observation for monitoring and further management. The patient was diagnosed with post-EGD bleeding, decompensated NAFLD, and portal hypertension. He was started on terlipressin 2 mg QDS IV and ciprofloxacin IV for 72 hours, after which the ciprofloxacin was switched to the oral route to complete the full five-day course. His blood tests during his admission are in Table 1.

**Table 1:** Blood tests during the patient’s five-day hospital admission ALP, alkaline phosphatase; ALT, alanine transaminase; PT, prothrombin time

	29/11/2022 (pre-admission)	12/12/2022 (Day 0, day of admission)	13/12/2022 (Day 1)	14/12/2022 (Day 2)	15/12/2022 (Day 3)	17/12/2022 (Day 5, day of discharge)	Reference range
Hemoglobin	138 g/L	135 g/L	120 g/L	122 g/L	119 g/L	125 g/L	130-180 g/L
Platelets	134x10^9^/L	117x10^9^/L	95x10^9^/L	101x10^9^/L	78x10^9^/L	100x10^9^/L	150-400x10^9^/L
Bilirubin	26 μmol/L	25 μmol/L	18 μmol/L	22 μmol/L	32 μmol/L	26 μmol/L	3-17 μmol/L
ALT	15 IU/L	Hemolyzed	15 IU/L	14 IU/L	13 IU/L	16 IU/L	3-40 IU/L
ALP	122 IU/L	Hemolyzed	102 IU/L	94 IU/L	81 IU/L	72 IU/L	30-100 IU/L
Albumin	31 g/L	33 g/L	33 g/L	35 g/L	37 g/L	31 g/L	35-50 g/L
PT	Not done	12.6 s	Not done	Not done	13.3 s	12.1 s	12.6-15.4 s

During his inpatient stay, he had an ascitic drain inserted, which drained approximately 10.5 L. His hemoglobin and blood pressure were stable throughout his admission. He was discharged on day 5 with oral carvedilol 3.125 mg OD and spironolactone 100 mg OD.

**Figure 3:**
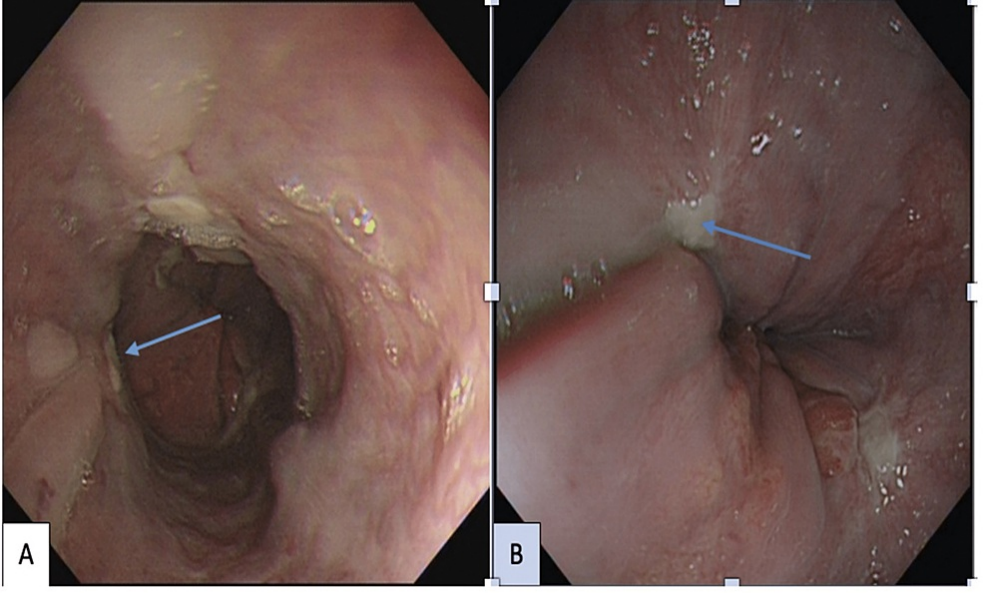
Repeat EGD two weeks later. (A) Post-banding ulcers with no active bleeding (arrow shows previous bleeding source). (B) Close-up of a post-banding ulcer EGD, esophagogastroduodenoscopy

Following a repeat EGD two weeks later, no active bleeding was detected; five post-banding ulcers were observed and one varix grade 1 was found (Figure 3A and Figure 3B).

**Figure 4:**
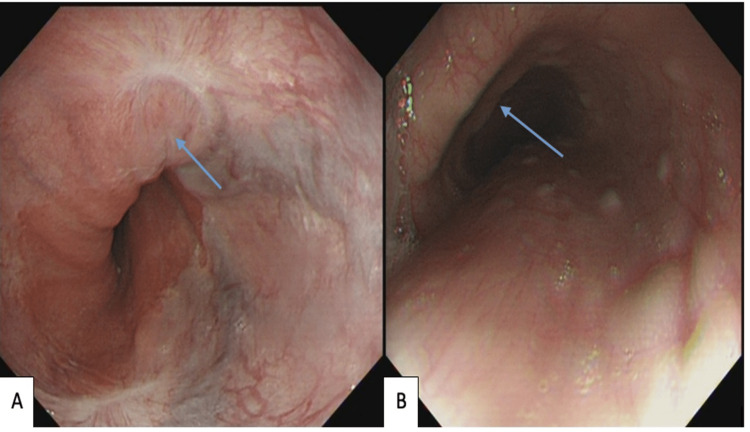
Follow-up EGD after two months. (A) Grade 1 esophageal varix. (B) Close-up of healed esophageal varix.

During a follow-up EGD after two months, two grade 1 esophageal varix was identified, without any high-risk stigmata. The varices were considered non-significant to warrant further banding (Figure 4A and Figure 4B).

## Discussion

Our case report is a unique example of using Purastat as an additional treatment option for patients experiencing an acute variceal bleed. Medical interventions such as vasoactive drugs have shown efficacy, and endoscopic interventions including band ligation and sclerotherapy have also been shown to be effective [8]. However, these conventional treatments have certain disadvantages that need to be considered.

Band ligation and sclerotherapy are commonly employed for variceal bleeding, but in some cases, they may not effectively control bleeding, as seen in our patient. Furthermore, sclerotherapy is typically reserved for smaller varices that are not amenable to band ligation. Sclerotherapy carries the risk of adverse events, including ulceration, and may require repeated sessions for complete eradication of the varices [9]. Removable stent placement, although effective in controlling bleeding, carries complications, as studies have reported adverse events related to stent placement, such as ulceration and stent migration [10]. The need for subsequent removal of the stent can also be challenging and carries its own complications [10].

Balloon tamponade is another option to achieve short-term hemostasis in patients with bleeding varices. However, it is associated with complications such as rebleeding upon balloon deflation, and its use is reserved for temporary stabilization of hemodynamically unstable patients until more definitive treatment can be instituted [11].

TIPS is a definitive treatment option for refractory variceal bleeding. However, it requires careful patient selection and rigorous assessment before consideration. Therefore, for refractory variceal bleeding cases, balloon tamponade or stent is the bridging option to TIPS [12].

In contrast, Purastat offers the unique advantage that it can be used in addition to standard medical and endoscopic interventions but has fewer complications associated with it than balloon tamponade or removable Danis stent insertion. It may prove useful in highly selected cases of refractory bleeding post variceal banding such as described in this case report. Additionally, Purastat can be used in conjunction with other therapeutics, allowing more flexibility in the management of bleeding cases. This feature sets it apart from the alternatives that may restrict the use of subsequent interventions after their application. 

Long-term management and follow-up are also important considerations for patients who have experienced an acute variceal bleed. These patients require ongoing monitoring to prevent rebleeding and manage complications such as portal hypertension [13]. In addition, lifestyle modifications such as alcohol cessation and weight loss may be necessary to improve outcomes [14]. 

## Conclusions

Overall, our case report highlights Purastat as a novel and promising additional treatment option for patients with an acute variceal bleed. Its safe application and ability to achieve rapid hemostasis make it a valuable tool in managing refractory bleeding cases. Further studies are warranted to fully elucidate the efficacy and potential advantages of Purastat compared to conventional treatments in acute variceal bleeding scenarios. Nevertheless, our case provides initial evidence of encouraging results obtained with Purastat and opens avenues for further exploration in this area.
